# Supplementary material for: Parallel Evolution at the Regulatory Base-Pair Level Contributes to Mammalian Interspecific Differences in Polygenic Traits
Source: Mol Biol Evol. 2024 Jul 29;41(8):msae157. doi: 10.1093/molbev/msae157 (PMC11321361; doi:10.1093/molbev/msae157)
Supplement: msae157_Supplementary_Data [file msae157_supplementary_data.zip › Parallel_Evolution_Supplement_Final.pdf]

Supplementary Information for

Parallel evolution at the regulatory base-pair level contributes to mammalian inter-specific differences in polygenic traits

Alexander S. Okamoto[1], Terence D. Capellini[1,2]

Corresponding authors:
Alexander S. Okamoto
Email: aokamoto@g.harvard.edu
Terence D. Capellini
Email: tcapellini@fas.harvard.edu

This PDF file includes:

- Supplementary Notes
  - Sources of variation
  - The characteristics of common human variation
  - Linkage disequilibrium and haplotype conservation
- Materials and Methods
  - Additional Body Mass Data for Zoonomia Species
- Supplementary References
- Figs S1 to S11
- Tables S1 to S4 Captions

Other supporting materials for this manuscript include the following:

Table 1 to 2, S1 to S4

Supplementary Notes

1. **Sources of variation.** If a given genomic position is to evolve in parallel in divergent lineages, the same variation must arise before it can be targeted by natural selection. For closely related species, this may be due to standing variation from the ancestral population as is the case in the parallel evolution of coat color in beach mice (Wooldridge et al. 2022). In the absence of standing variation, recurrent evolution at a given position is required. While such an event may be rare, this is unlikely to be limiting over evolutionary timescales. A simple calculation multiplying the current human population by the number of *de novo* mutations per person (Acuna-Hidalgo, Veltman, and Hoischen 2016) and dividing by the number of positions in the haploid genome suggests that every possible position should be mutated in at least 100 individuals. While mutations are obviously not evenly distributed across the genome, with the real probability of a given mutation governed by the local mutation rate as well as biochemical limitation such as the increased likelihood of transition to transversion events, over many generations and large population sizes, most variants likely appear at least a few times in each lineage. Mutational hotspots are especially likely to generate the variants necessary for parallel evolution at a specific locus (Xie et al. 2019).

2. **The characteristics of common human variation.** Common genetic variants in modern humans (defined here as variants with a minor allele frequency > 0.01 in the UK Biobank) have a few key properties which are advantageous for this study.

First off, millions of human genomes have been sequenced, providing a vast catalog of common variants and their frequencies in the target population (Bycroft et al. 2018). Second, since common human variants are found in large numbers of people, they can only be mildly deleterious since mutations that are embryonic lethal or otherwise cause severe disease with high penetrance are extremely unlikely to persist in the population at this frequency threshold. Thirdly, since much of this genetic information is paired with detailed phenotypic data, thousands of SNPs have been associated with phenotypic traits through GWAS. Therefore, by evaluating the position of common human SNPs with phenotypic associations in the genomes of other mammals, there is an elevated chance that the SNP positions are more biologically relevant and tolerant of mutation than random genomic positions.

3. **Linkage disequilibrium and haplotype conservation.** While haplotype structure poses a substantial challenge when comparing humans, LD is rapidly disrupted over evolutionary timescales. For example, humans and chimpanzees only have 125 genomic regions in which the same haplotypes are still segregating (Leffler et al. 2013). Over longer evolutionary timescales, haplotypes are likely to have been lost due to recombination, fixed in the lineages, or acquired additional mutations to form novel haplotypes. Therefore, if some set of variants in the genomes of humans and chimpanzees have the same biological function, those variants might be more readily identified as causative by comparing across species than within species because linked neutral variants in both species are unlikely to be shared

due to divergence in haplotype structure. This argument can be extended to more distantly related species with the caveat that less variants are expected to have the same biological functions due to increasing regulatory divergence between more distantly related species. Conserved regions are under evolutionary constraint so mutations in these regions are more likely to have functional effects. Indeed, SNPs associated with human diseases in GWAS are 1.37-fold enriched in mammalian constrained regions (Lindblad-Toh et al. 2011). Thus, the genomes of other species can potentially be used to identify causative variants in humans that lie within important regulatory regions.

Extended Materials and Methods

Additional Body Mass Data for Zoonomia Species

Species without body mass data in the Pantheria Database (K. E. Jones et al. 2009) were supplemented with the additional information listed below. Where only a body mass range was available, the mean was taken within and between sexes.

1. *Allactaga bullata*, data unavailable
2. *Balaenoptera bonaerensis*, male used since only pregnant female data available, 8,350,000 g (Konishi 2006)
3. *Bos indicus*, 280,000 g (Abdelhadi and Babiker 2009)
4. *Bos mutus,* 546,250 g (Wilson and Mittermeier 2011)
5. *Camelus ferus,* 475,000 g (Wilson and Mittermeier 2011)
6. *Canis lupus familiaris,* given the variability of dog breed sizes, dogs were excluded from the analysis.
7. *Capra aegagrus*, 53,750 g (Wilson and Mittermeier 2011)
8. *Ceratotherium simum cottoni*, 1,900,000 g (Hillman-Smith et al. 1986)
9. *Chlorocebus sabaeus,* 3,975 g (Mittermeier, Rylands, and Wilson 2013)
10. *Cricetulus griseus,* 40 g (Wilson, Lacher, and Mittermeier 2017)
11. *Crocidura indochinensis*, data unavailable
12. *Ellobius lutescens*, 71 g (Wilson, Lacher, and Mittermeier 2017)
13. *Equus przewalskii,* 250,000 g (Wilson and Mittermeier 2011)
14. *Eubalaena japonica*, 57,595,750 g (Christiansen et al. 2019)

15. *Eulemur flavifrons,* 1,850 g (Mittermeier, Rylands, and Wilson 2013)
16. *Fukomys damarensis*, 153.25 g (Wilson, Lacher, and Mittermeier 2016)
17. *Galeopterus variegatus,* 1,450 g (Wilson and Mittermeier 2018)
18. *Ictidomys tridecemlineatus*, 190 g (Wilson, Lacher, and Mittermeier 2016)
19. *Murina feae,* 4.8 g (Wilson and Mittermeier 2019)
20. *Mus pahari*, 24 g (Wilson, Lacher, and Mittermeier 2017)
21. *Myotis davidii,* 5.95 g (Wilson and Mittermeier 2019)
22. *Nannospalax galili, ~N. ehrenbergi,* 162.5 g (Wilson, Lacher, and Mittermeier 2017)
23. *Neomonachus schauinslandi,* 204,000 g (Wilson and Mittermeier 2014)
24. *Neophocaena asiaeorientalis*, 56,000 g (Wilson and Mittermeier 2014)
25. *Piliocolobus tephrosceles*, males only, 9,700 g (Mittermeier, Rylands, and Wilson 2013)
26. *Rhinolophus sinicus*, 9.9 g (Wilson and Mittermeier 2019)
27. *Spermophilus dauricus,* 223.8 g (Wilson, Lacher, and Mittermeier 2016)
28. *Spilogale gracilis,* 492.5 g (K. L. Jones, Van Vuren, and Crooks 2008)
29. *Tonatia saurophila*, 28.56 g (López-Aguirre et al. 2021)
30. *Tupaia chinensis*, 125 g (Yao 2017)
31. *Uropsilus gracilis*, data unavailable
32. *Vicugna pacos*, 44,400 g (Pérez et al. 2016)

Supplementary References:

Abdelhadi, OMA, and SA Babiker. 2009. “Prediction of Zebu Cattle Live Weight Using Live Animal Measurements.” *Livestock Research for Rural Development* 21 (8). http://www.lrrd.org/lrrd21/8/abde21133.htm.

Acuna-Hidalgo, Rocio, Joris A. Veltman, and Alexander Hoischen. 2016. “New Insights into the Generation and Role of de Novo Mutations in Health and Disease.” *Genome Biology* 17 (1): 241. https://doi.org/10.1186/s13059-016-1110-1.

Bycroft, Clare, Colin Freeman, Desislava Petkova, Gavin Band, Lloyd T. Elliott, Kevin Sharp, Allan Motyer, et al. 2018. “The UK Biobank Resource with Deep Phenotyping and Genomic Data.” *Nature* 562 (7726): 203–9. https://doi.org/10.1038/s41586-018-0579-z.

Christiansen, Fredrik, Mariano Sironi, Michael J. Moore, Matías Di Martino, Marcos Ricciardi, Hunter A. Warick, Duncan J. Irschick, Robert Gutierrez, and Marcela M. Uhart. 2019. “Estimating Body Mass of Free-living Whales Using Aerial Photogrammetry and 3D Volumetrics.” Edited by Graziella Iossa. *Methods in Ecology and Evolution* 10 (12): 2034–44. https://doi.org/10.1111/2041-210X.13298.

Hillman-Smith, A. K. K., N. Owen-Smith, J. L. Anderson, A. J. Hall-Martin, and J. P. Selaladi. 1986. “Age Estimation of the White Rhinoceros (Ceratotherium Simum).” *Journal of Zoology* 210 (3): 355–77. https://doi.org/10.1111/j.1469-7998.1986.tb03639.x.

Jones, Kate E., Jon Bielby, Marcel Cardillo, Susanne A. Fritz, Justin O’Dell, C. David L. Orme, Kamran Safi, et al. 2009. “PanTHERIA: A Species-Level Database of Life History, Ecology, and Geography of Extant and Recently Extinct Mammals.” Edited

by W. K. Michener. *Ecology* 90 (9): 2648–2648. https://doi.org/10.1890/08-1494.1.
Jones, Krista L., Dirk H. Van Vuren, and Kevin R. Crooks. 2008. “Sudden Increase in a Rare Endemic Carnivore: Ecology of the Island Spotted Skunk.” *Journal of Mammalogy* 89 (1): 75–86. https://doi.org/10.1644/07-MAMM-A-034.1.
Konishi, Kenji. 2006. “Characteristics of Blubber Distribution and Body Condition Indicators for Antarctic Minke Whales (Balaenoptera Bonaerensis).” *Mammal Study* 31 (1): 15–22. https://doi.org/10.3106/1348-6160(2006)31[15:COBDAB]2.0.CO;2BDAB]2.0.CO;2.
Leffler, E. M., Z. Gao, S. Pfeifer, L. Segurel, A. Auton, O. Venn, R. Bowden, et al. 2013. “Multiple Instances of Ancient Balancing Selection Shared Between Humans and Chimpanzees.” *Science* 339 (6127): 1578–82. https://doi.org/10.1126/science.1234070.
Lindblad-Toh, Kerstin, Manuel Garber, Or Zuk, Michael F. Lin, Brian J. Parker, Stefan Washietl, Pouya Kheradpour, et al. 2011. “A High-Resolution Map of Human Evolutionary Constraint Using 29 Mammals.” *Nature* 478 (7370): 476–82. https://doi.org/10.1038/nature10530.
López-Aguirre, Camilo, Nicholas J. Czaplewski, Andrés Link, Masanaru Takai, and Suzanne J. Hand. 2021. “Dietary and Body-Mass Reconstruction of the Miocene Neotropical Bat Notonycteris Magdalenensis (Phyllostomidae) from La Venta, Colombia.” *Paleobiology*, July, 1–17. https://doi.org/10.1017/pab.2021.21.
Mittermeier, RA, AB Rylands, and DE Wilson, eds. 2013. *Handbook of the Mammals of the World. Vol. 3. Primates*. Barcelona: Lynx Edicions.
Pérez, William, Horst Erich König, Hassen Jerbi, and Marcus Clauss. 2016.

"Macroanatomical Aspects of the Gastrointestinal Tract of the Alpaca (Vicugna Pacos Linnaeus, 1758) and Dromedary (Camelus Dromedarius Linnaeus, 1758)." *Vertebrate Zoology* 66 (3): 419–25.

Wilson, DE, TE Jr. Lacher, and RA Mittermeier, eds. 2016. *Handbook of the Mammals of the World. Vol. 6. Lagomorphs and Rodents 1.* Barcelona: Lynx Edicions.

Wilson, DE, TE Jr. Lacher, and RA Mittermeier, eds. 2017. *Handbook of the Mammals of the World. Vol. 7. Rodents 2.* Barcelona: Lynx Edicions.

Wilson, DE, and RA Mittermeier, eds. 2011. *Handbook of the Mammals of the World. Vol. II. Hoofed Mammals*. Barcelona: Lynx Edicions.

Wilson, DE, and RA Mittermeier, eds. 2014. *Handbook of the Mammals of the World. Vol. 4. Sea Mammals*. Barcelona: Lynx Edicions.

Wilson, DE, and RA Mittermeier, eds. 2018. *Handbook of the Mammals of the World. Vol. 8. Insectivores, Sloths, and Colugos*. Barcelona: Lynx Edicions.

Wilson, DE, and RA Mittermeier, eds. 2019. *Handbook of the Mammals of the World. Vol. 9. Bats*. Barcelona: Lynx Edicions.

Wooldridge, T. Brock, Andreas F. Kautt, Jean-Marc Lassance, Sade McFadden, Vera S. Domingues, Ricardo Mallarino, and Hopi E. Hoekstra. 2022. "An Enhancer of Agouti Contributes to Parallel Evolution of Cryptically Colored Beach Mice." *Proceedings of the National Academy of Sciences* 119 (27). https://doi.org/10.1073/pnas.2202862119.

Xie, Kathleen T., Guliang Wang, Abbey C. Thompson, Julia I. Wucherpfennig, Thomas E. Reimchen, Andrew D. C. MacColl, Dolph Schluter, Michael A. Bell, Karen M. Vasquez, and David M. Kingsley. 2019. "DNA Fragility in the Parallel Evolution of

Pelvic Reduction in Stickleback Fish.” *Science* 363 (6422): 81–84. https://doi.org/10.1126/science.aan1425.

Yao, Yong-Gang. 2017. “Creating Animal Models, Why Not Use the Chinese Tree Shrew (Tupaia Belangeri Chinensis)?” *Zoological Research* 38 (3): 118–26. https://doi.org/10.24272/j.issn.2095-8137.2017.032.

Supplementary Figures

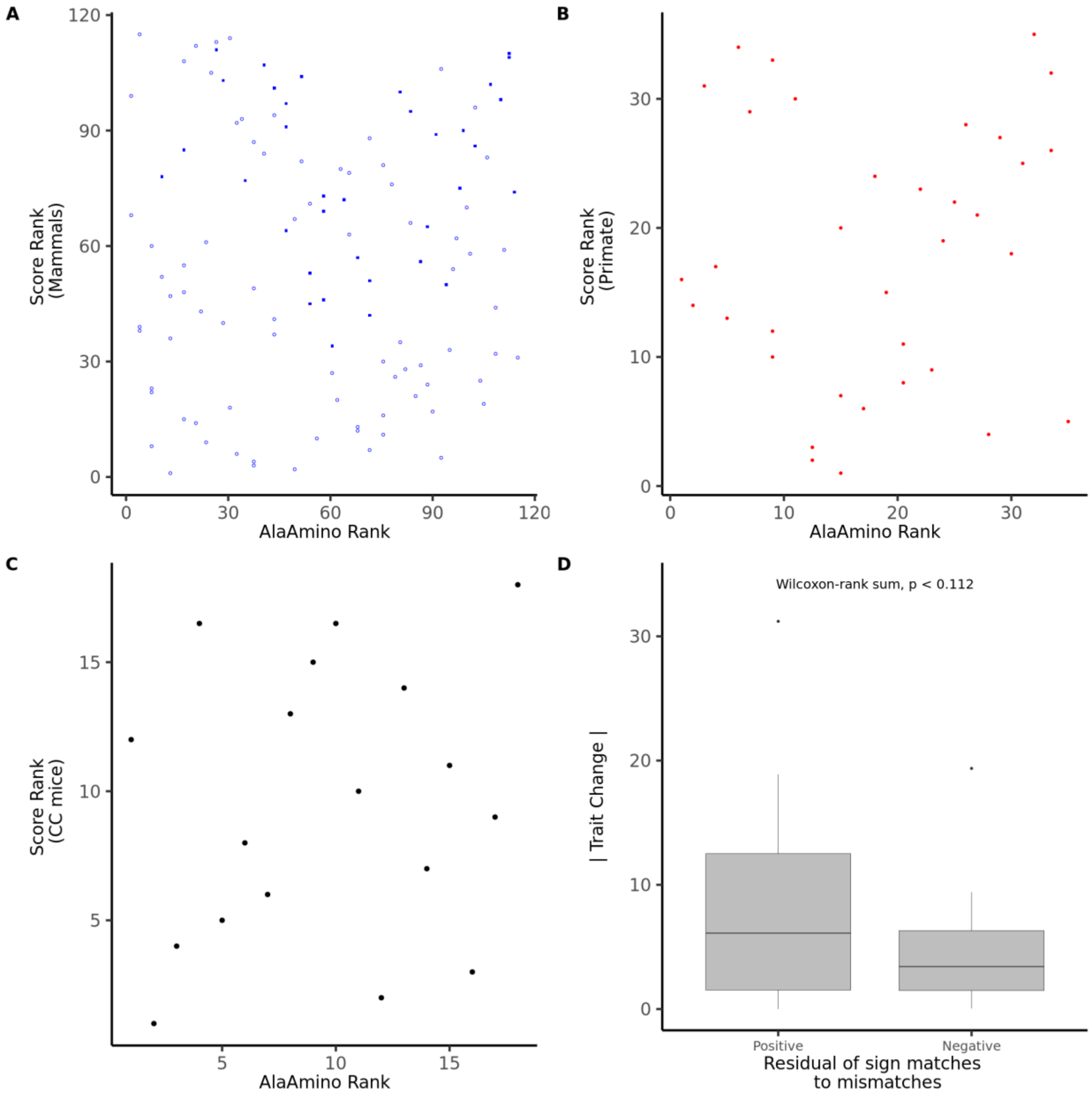

Figure S1. PGLS regression of alanine aminotransferase rank against the genomic score computed using mammal conserved SNP positions (A) or primate conserved SNP positions (B). In A, closed squares represent primate species, open circle represent non-primate mammals. (C) Linear regression of alanine aminotransferase rank against genomic score computed for collaborative cross mouse lines. (D) Comparison of absolute magnitude of alanine aminotransferase change for internal branches with a positive

residual of matches (allele effect direction matches phenotypic change) to mismatches compared to those with a negative change. Regression lines only show for significant relationships.

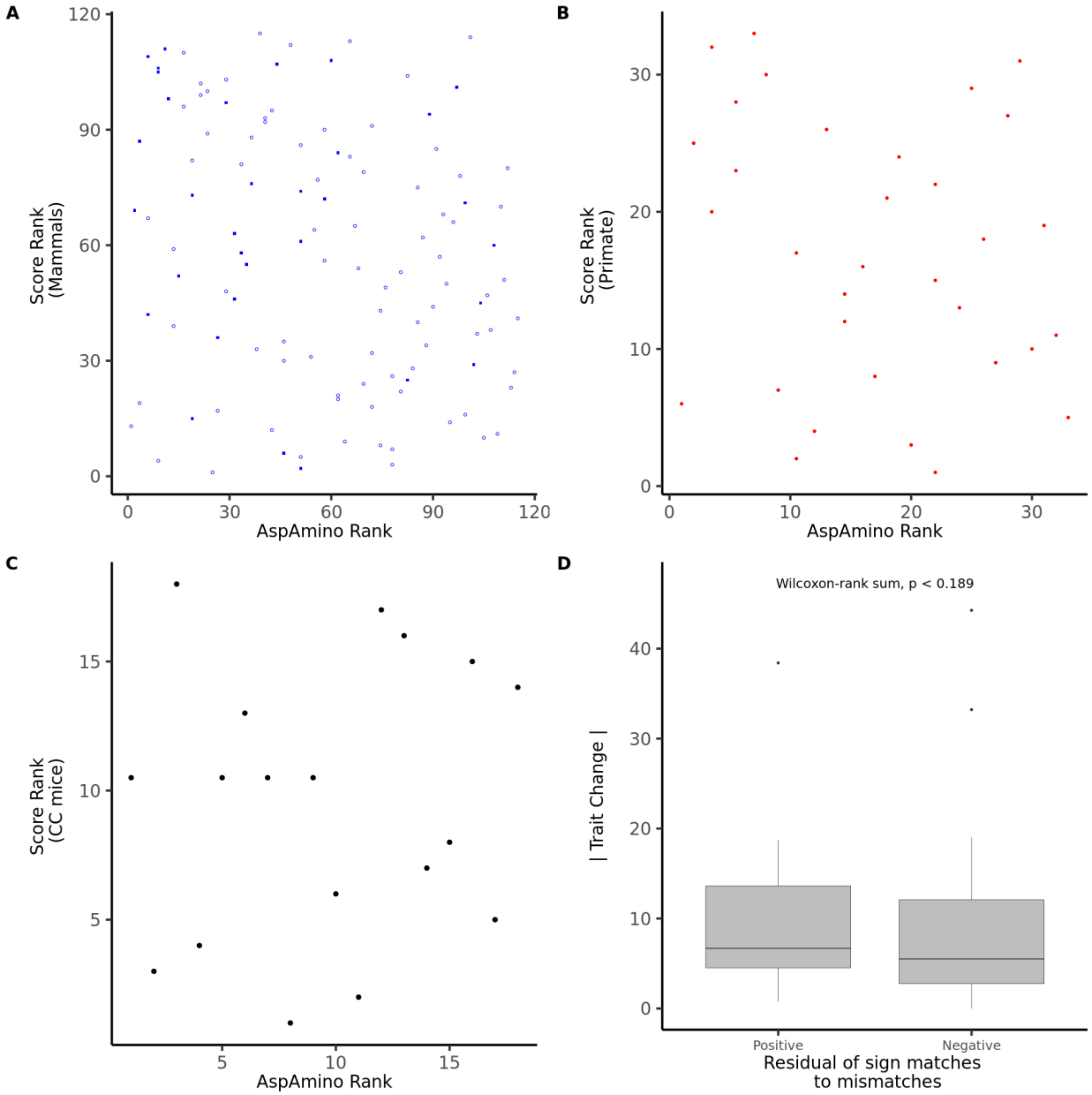

Figure S2. PGLS regression of aspartate aminotransferase rank against the genomic score computed using mammal conserved SNP positions (A) or primate conserved SNP positions (B). In A, closed squares represent primate species, open circle represent non-primate mammals. (C) Linear regression of aspartate aminotransferase rank against genomic score computed for collaborative cross mouse lines. (D) Comparison of absolute magnitude of aspartate aminotransferase change for internal branches with a positive residual of matches (allele effect direction matches phenotypic change) to mismatches compared to those with a negative change. Regression lines only show for significant relationships.

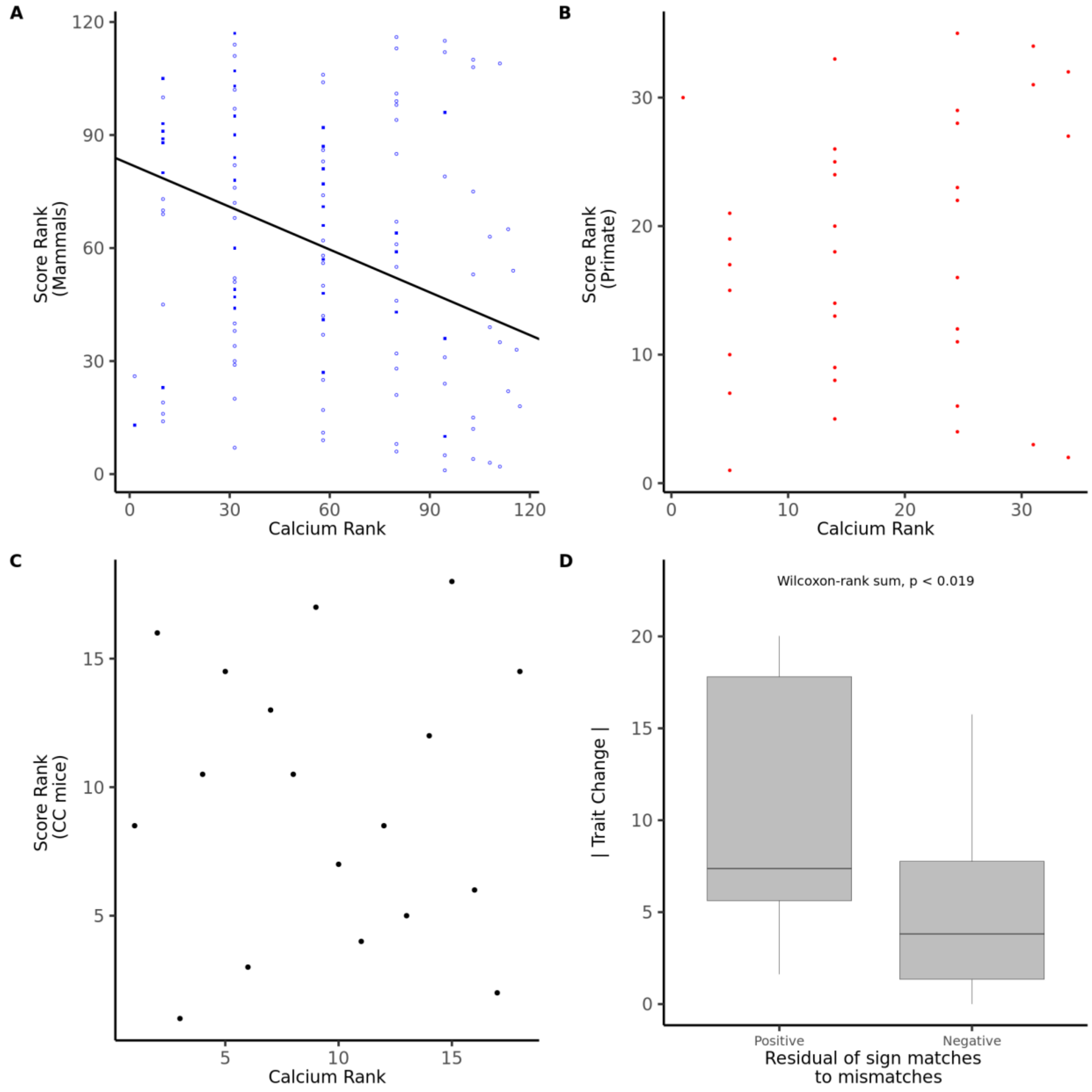

Figure S3. PGLS regression of calcium rank against the genomic score computed using mammal conserved SNP positions (A) or primate conserved SNP positions (B). In A, closed squares represent primate species, open circle represent non-primate mammals. (C) Linear regression of calcium rank against genomic score computed for collaborative cross mouse lines. (D) Comparison of absolute magnitude of calcium change for internal branches with a positive residual of matches (allele effect direction matches

phenotypic change) to mismatches compared to those with a negative change. Regression lines only show for significant relationships.

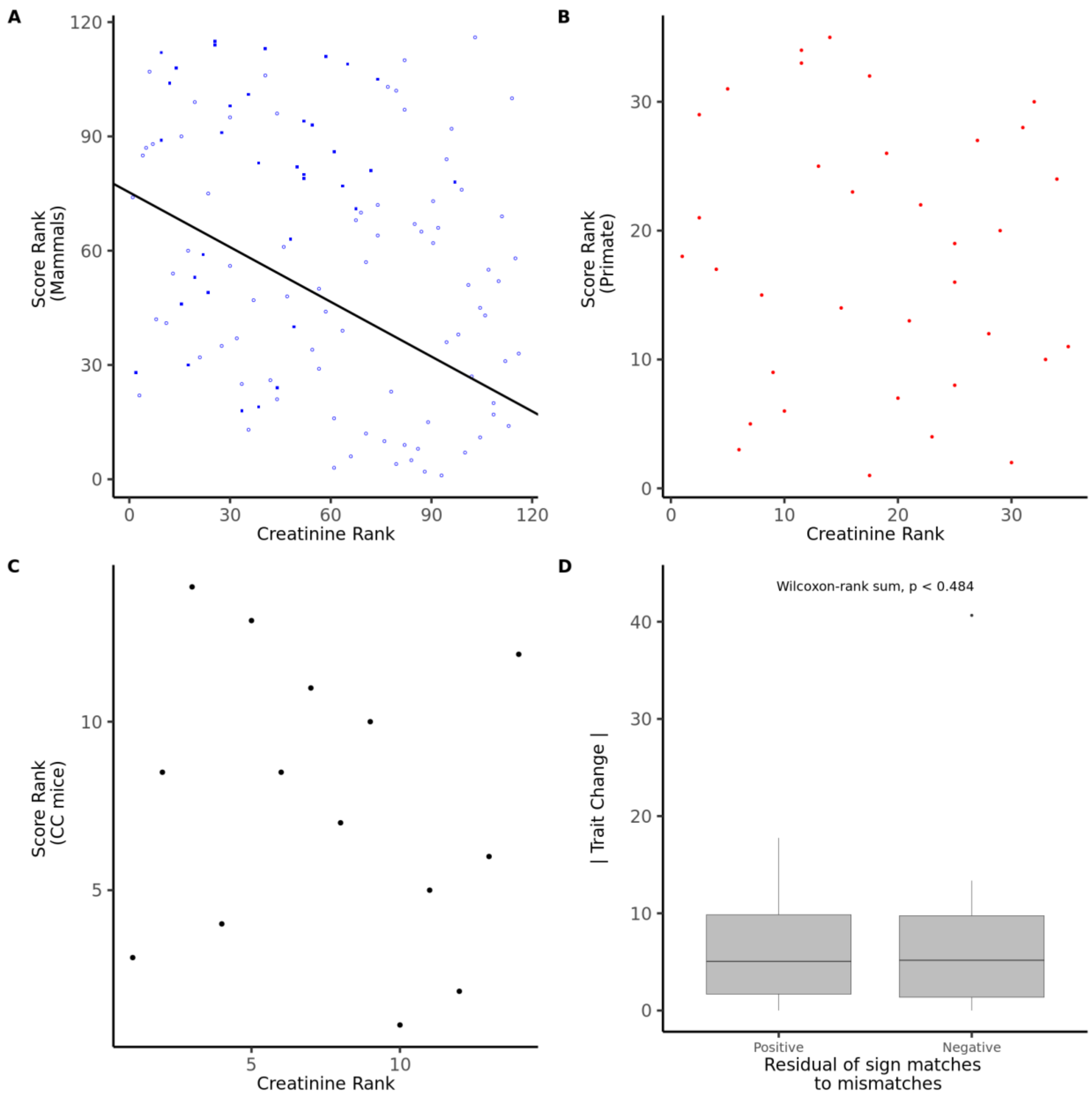

Figure S4. PGLS regression of creatinine rank against the genomic score computed using mammal conserved SNP positions (A) or primate conserved SNP positions (B). In A, closed squares represent primate species, open circle represent non-primate mammals. (C) Linear regression of creatinine rank against genomic score computed for collaborative cross mouse lines. (D) Comparison of absolute magnitude of creatinine change for internal branches with a positive residual of matches (allele effect direction matches phenotypic change) to mismatches compared to those with a negative change. Regression lines only show for significant relationships.

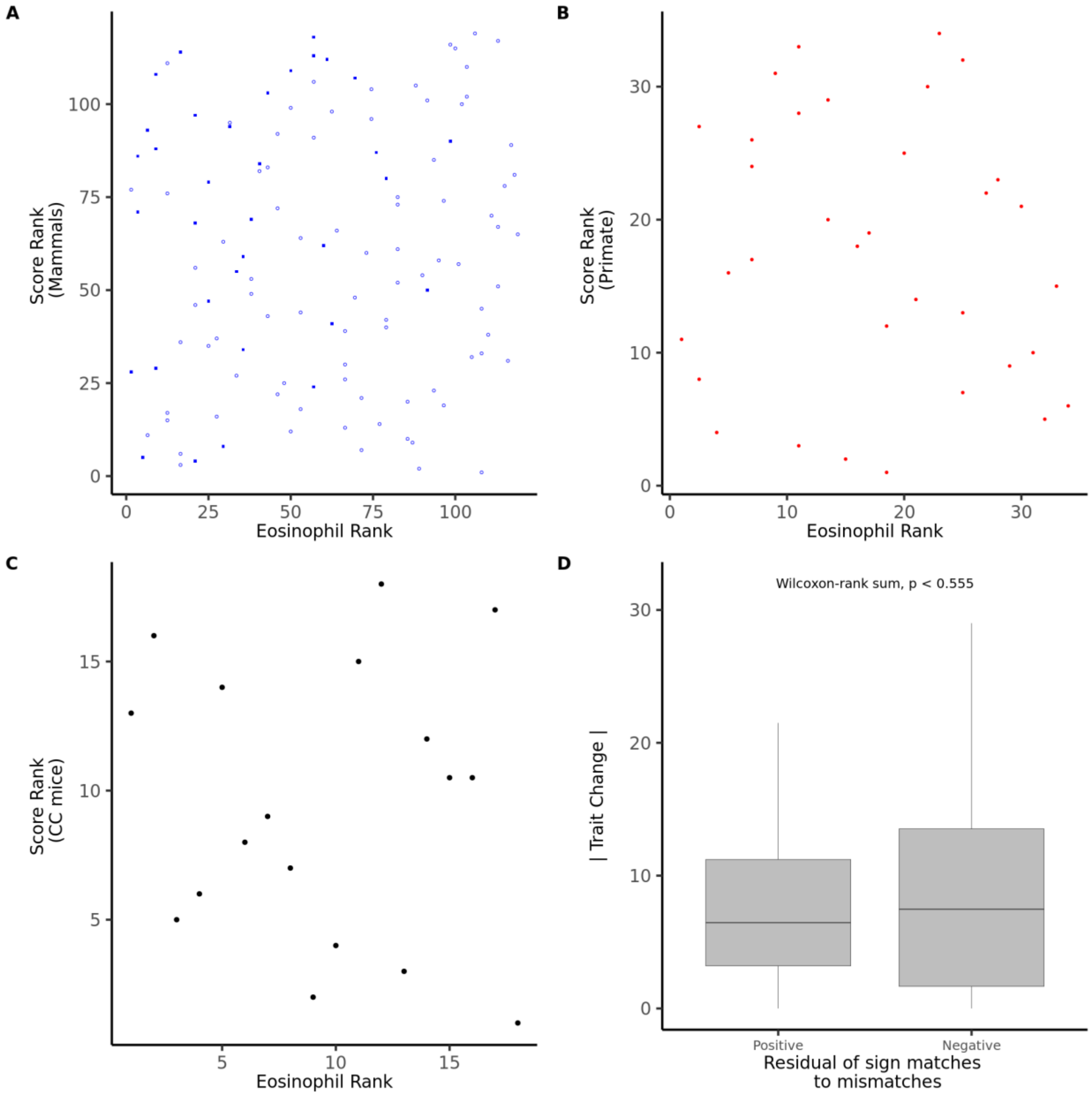

Figure S5. PGLS regression of eosinophil rank against the genomic score computed using mammal conserved SNP positions (A) or primate conserved SNP positions (B). In A, closed squares represent primate species, open circle represent non-primate mammals. (C) Linear regression of eosinophil rank against genomic score computed for collaborative cross mouse lines. (D) Comparison of absolute magnitude of eosinophil change for internal branches with a positive residual of matches (allele effect direction matches phenotypic change) to mismatches compared to those with a negative change. Regression lines only show for significant relationships.

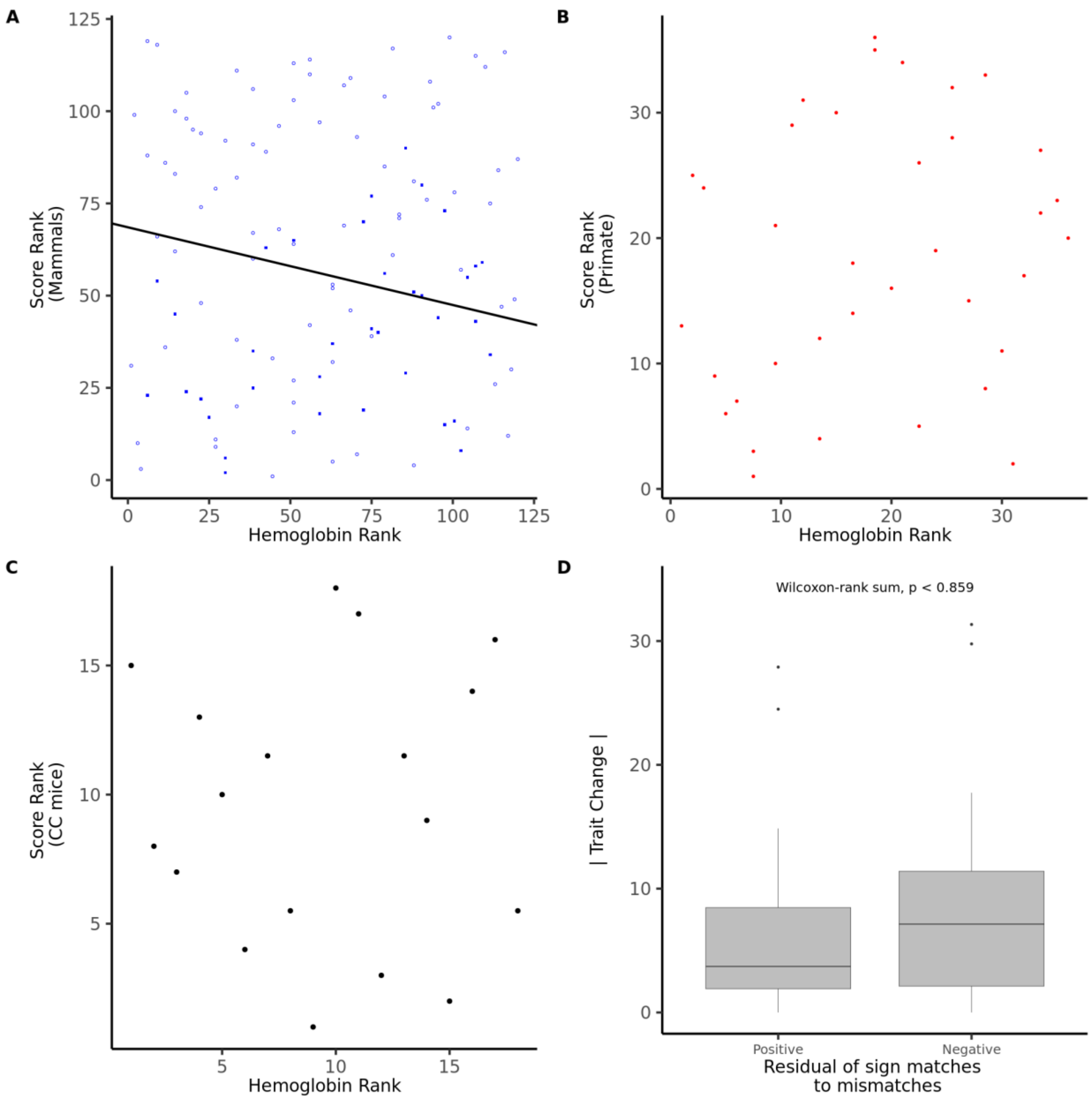

Figure S6. PGLS regression of hemoglobin rank against the genomic score computed using mammal conserved SNP positions (A) or primate conserved SNP positions (B). In A, closed squares represent primate species, open circle represent non-primate mammals. (C) Linear regression of hemoglobin rank against genomic score computed for collaborative cross mouse lines. (D) Comparison of absolute magnitude of hemoglobin change for internal branches with a positive residual of matches (allele effect direction matches phenotypic change) to mismatches compared to those with a negative change. Regression lines only show for significant relationships.

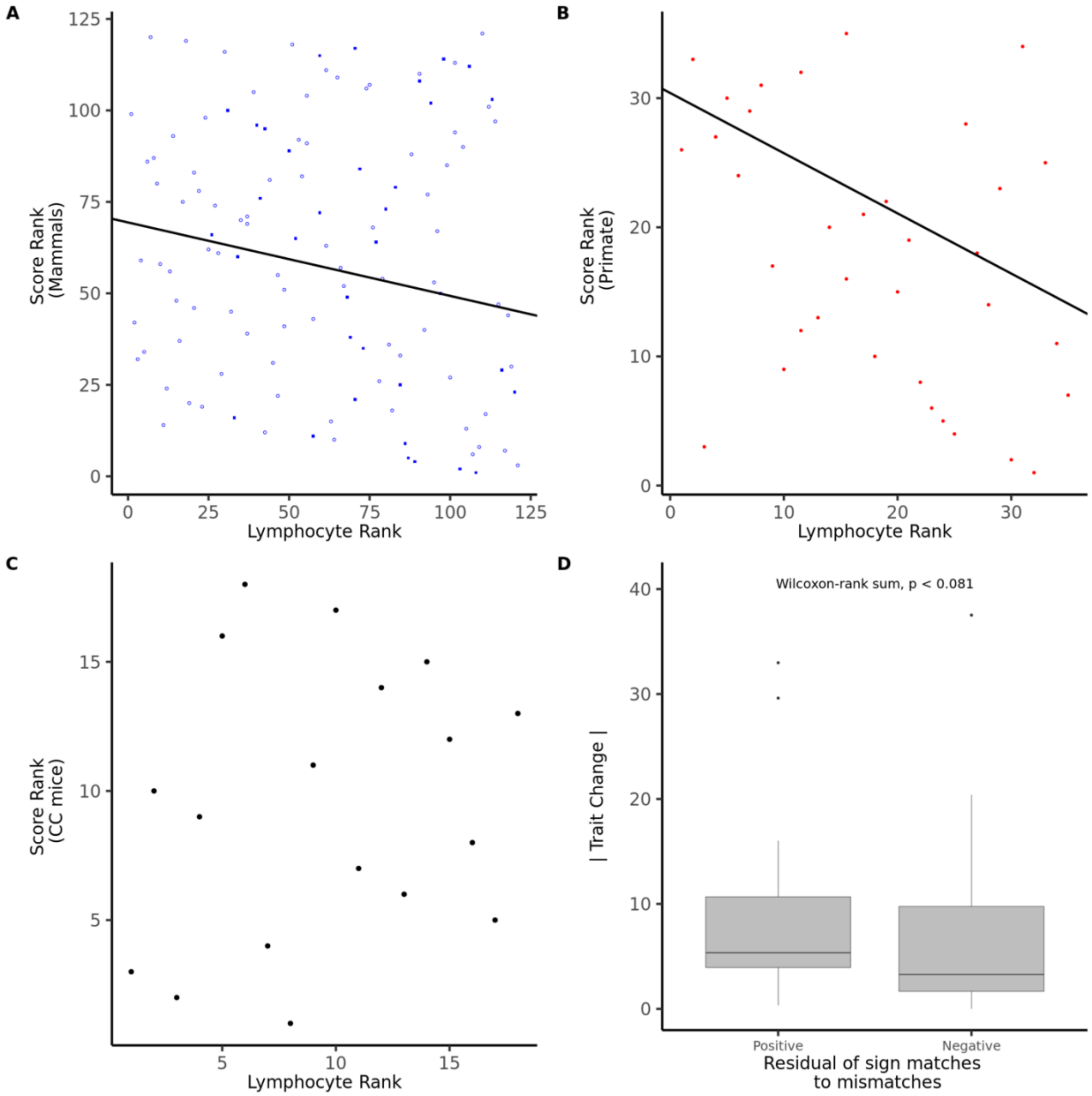

Figure S7. PGLS regression of lymphocyte rank against the genomic score computed using mammal conserved SNP positions (A) or primate conserved SNP positions (B). In A, closed squares represent primate species, open circle represent non-primate mammals. (C) Linear regression of lymphocyte rank against genomic score computed for collaborative cross mouse lines. (D) Comparison of absolute magnitude of lymphocyte change for internal branches with a positive residual of matches (allele effect direction matches phenotypic change) to mismatches compared to those with a negative change. Regression lines only show for significant relationships.

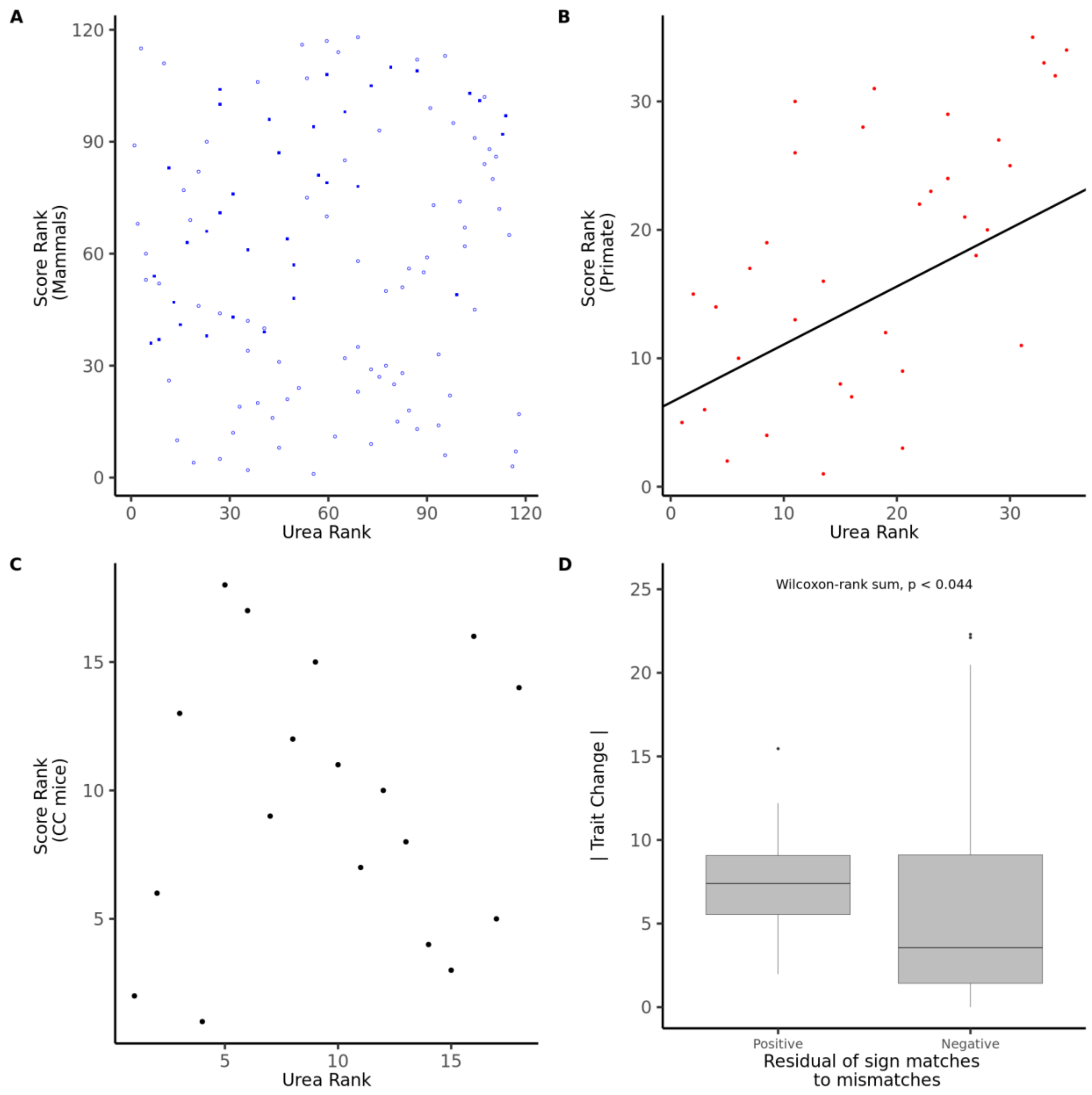

Figure S8. PGLS regression of urea rank against the genomic score computed using mammal conserved SNP positions (A) or primate conserved SNP positions (B). In A, closed squares represent primate species, open circle represent non-primate mammals. (C) Linear regression of urea rank against genomic score computed for collaborative cross mouse lines. (D) Comparison of absolute magnitude of urea change for internal branches with a positive residual of matches (allele effect direction matches phenotypic change) to mismatches compared to those with a negative change. Regression lines only show for significant relationships.

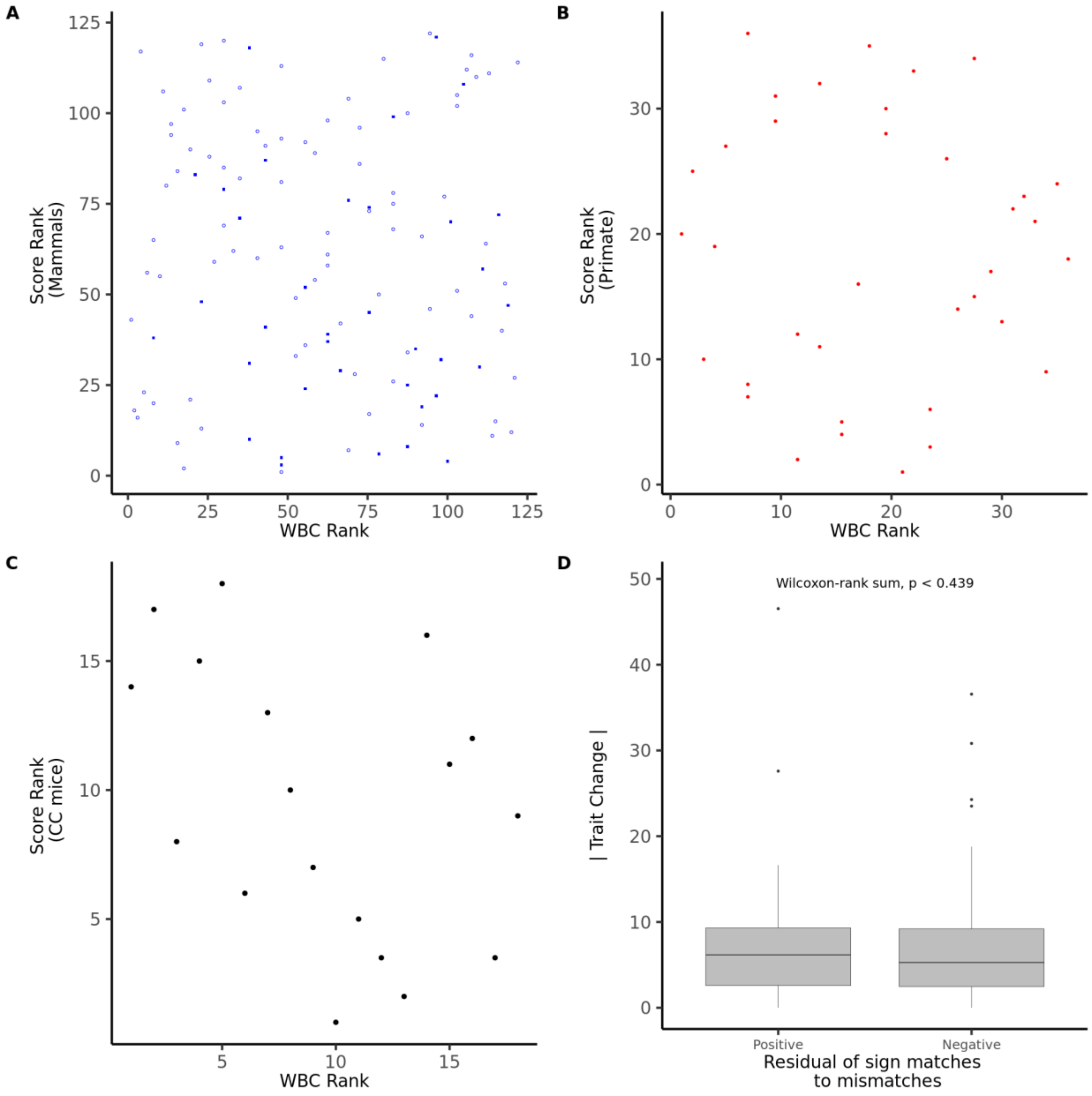

Figure S9. PGLS regression of white blood cell (WBC) rank against the genomic score computed using mammal conserved SNP positions (A) or primate conserved SNP positions (B). In A, closed squares represent primate species, open circle represent non-primate mammals. (C) Linear regression of white blood cell rank against genomic score computed for collaborative cross mouse lines. (D) Comparison of absolute magnitude of white blood cell change for internal branches with a positive residual of matches

(allele effect direction matches phenotypic change) to mismatches compared to those with a negative change. Regression lines only show for significant relationships.

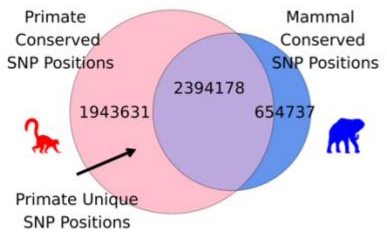

Figure S10. Venn diagram showing relative number of biallelic human SNP positions alignable to at least 75% of all mammals (blue circle, represented by the African elephant) or 85% of all primates (red circle, represented by the ring-tailed lemur). Positions which are uniquely alignable in primates are shown in pink. Silhouettes are from PhyloPic.

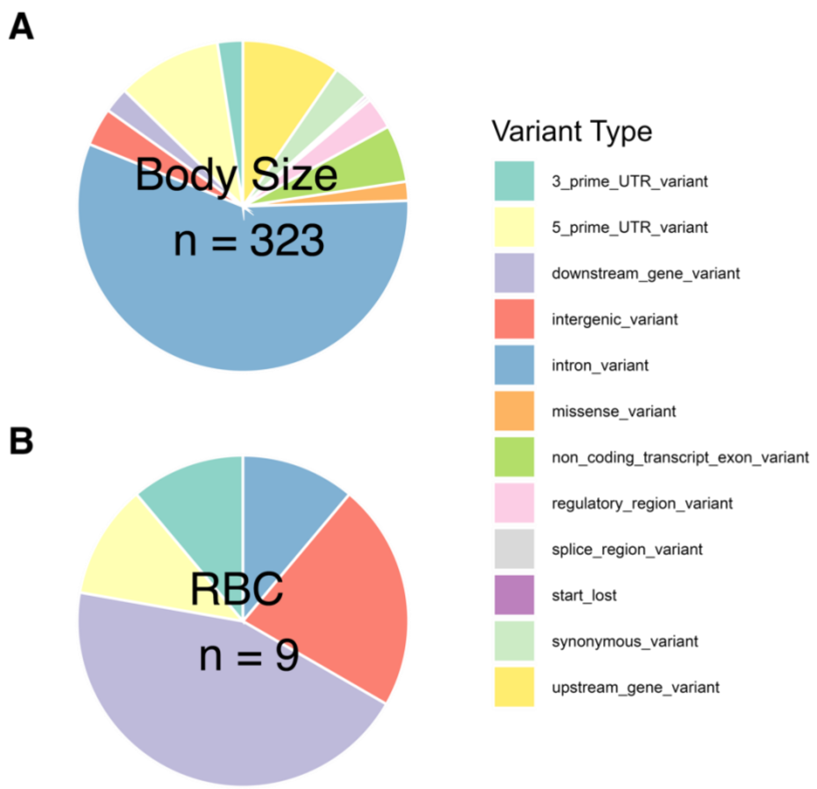

Figure S11. Types of putative causal variants overlapping conserved candidate *cis*-regulatory elements for body size (A) and red blood cell count (B).

Supplementary Tables

Table S1: Extended test results for each trait. Includes results for the PGLS regressions for eleven traits using genomic scores calculated based on SNP positions conserved in mammals (blue), primates (red), or primates but not other mammals (pink). The number of species (n) for which genomic and phenotypic information is listed for each comparison type along with the number of GWAS significant SNPs, the sign of the relationship slope, and *P*-values determined by PGLS and after permulations. *P*-values are shown for a Wilcoxon rank-sum test of enriched trait change along internal branches with positive residuals of trait matches to mismatches and for a Spearman correlation of genomic scores and trait rank matches in eighteen collaborative cross mice strains.

Table S2: Candidate causative variant information for red blood count. Table include columns for (1) the human RSID (2), the trait, (3) the human genomic position (hg38), (4) the human reference and alternate alleles, (5) the direction of effect of the alternate allele, (6) the Wilcoxon P-value for the association of each allele with the trait across the mammals investigated in this study, (7) the PhyloP score for a range of 21 BPs centered around the SNP position, (8) the variant type, (9) syntenic genes within a 1 MB window centered on the SNP in human, macaque, dog, cattle, and mouse, (10) genes for which this position is a cis-eQTL based on human blood samples, and (11) predicted TF motifs binding around this SNP in both human and mouse, (13-252) aligned allele for all species used in the study which match a human allele, (253) total number of species with alignments at this position, (254) number of alignments to human reference allele, (255) number of alignments to human alternative allele, (256) *P*-value for Wilcoxon rank-sum test for phenotype rank by allelic match, (257) mean phenotype rank of species with the reference allele, (258) mean phenotype rank of species with the alternate allele, and (259) Wilcoxon test W statistic.

Table S3: Candidate causative variant information for body size. Table include columns for (1) the human RSID (2), the trait, (3) the human genomic position (hg38), (4) the human reference and alternate alleles, (5) the direction of effect of the alternate allele, (6) the Wilcoxon P-value for the association of each allele with the trait across the mammals investigated in this study, (7) the PhyloP score for a range of 21 BPs centered around the SNP position, (8) the variant type, (9) syntenic genes within a 1 MB window centered

on the SNP in human, macaque, dog, cattle, and mouse, (10) genes for which this position is a cis-eQTL based on human blood samples, and (11) predicted TF motifs binding around this SNP in both human and mouse, (13-252) aligned allele for all species used in the study which match a human allele, (253) total number of species with alignments at this position, (254) number of alignments to human reference allele, (255) number of alignments to human alternative allele, (256) *P*-value for Wilcoxon rank-sum test for phenotype rank by allelic match, (257) mean phenotype rank of species with the reference allele, (258) mean phenotype rank of species with the alternate allele, and (259) Wilcoxon test W statistic.

Table S4: Resources used in this project. Lists the various datasets and computation resources used in this project and any relevant accession information.
